# Apophyseal Injuries of the Lower Extremities in Male Adolescent Football Players From a Single Academy: A One‐Season Prospective Cohort Study

**DOI:** 10.1155/tsm2/4422014

**Published:** 2026-10-16

**Authors:** Simen Rygh, Thor Einar Andersen, Joar Harøy

**Affiliations:** ^1^ Department of Sports Medicine at The Norwegian School of Sports Sciences Oslo Sports Trauma Research Center Oslo Norway; ^2^ Norwegian FA’s Sports Medicine Center Oslo Norway

## Abstract

**Background:**

Youth football players experience a high incidence of injuries, particularly apophyseal injuries in the lower extremities. However, the prevalence and characteristics of these injuries in male adolescent academy players remain inadequately documented.

**Objective:**

To investigate the prevalence, incidence, and characteristics of apophyseal injuries in the lower extremities among male adolescent academy football players over one season.

**Design:**

One‐season prospective cohort study.

**Participants:**

Fifty‐eight Norwegian male adolescent academy football players (aged 12–19 years) were investigated during the 2022 football season.

**Methods:**

Weekly self‐reports of health problems were collected using the Oslo Sports Trauma Research Center Questionnaire on Health Problems (OSTRC‐H2). Clinical examinations were performed to diagnose apophyseal injuries.

**Results:**

The average weekly prevalence of self‐reported health problems was 37%, while the average weekly prevalence of clinically diagnosed apophyseal injuries in the lower extremities was 12%. The incidence rate for these injuries was 4.6 per 1.000 h of exposure. Pubic apophysitis was the most common injury (32% of diagnosed cases). Most injuries (77%) had a gradual onset, with 81% occurring during or directly after training sessions.

**Conclusions:**

Apophyseal injuries represent a significant health problem in male youth football players, with pubic apophysitis being the most prevalent condition. These findings highlight the need for improved awareness, early detection, and targeted injury prevention strategies in youth football.

## 1. Introduction

Youth academy football players experience higher injury rates compared to senior players [1–3]. In a large study following 301 players over four seasons, 1111 time‐loss injuries in high‐level male youth footballers were documented [4]. The overall injury incidence was 12.0 per 1.000 h, with a burden of 255 days per 1.000 h, and apophyseal injuries were most common among younger players [4].

Physeal injuries are unique in adolescents, and they involve damage to the growth plate, with or without epiphyseal or apophyseal involvement [5–8]. Epiphyseal injuries occur near the ends of long bones and can disrupt growth, while apophyseal injuries involve the muscle insertion sites on immature skeletons secondary officiation centers [6, 8, 9]. The hip, groin, and pelvis regions represent the most frequently affected site for apophyseal injuries overall with the anterior inferior iliac spine (AIIS) as the most commonly reported [9–11]. Other studies, however, identified Osgood–Schlatter’s disease and Sever’s disease as the most prevalent [12, 13].

While previous studies on apophyseal injuries in adolescents predominantly have used a time‐loss injury definition, this approach may underestimate the true burden of these injuries that do not result in the absence from participation. The any‐physical‐complaint injury definition has been shown to be more sensitive in capturing all complaints, including those that may not lead to time‐loss but still impact pain experience, well‐being, participation, training volume, and performance [14–16]. To our knowledge and despite their significance in adolescent players, no studies have reported the weekly prevalence of apophyseal injuries.

This knowledge gap highlights the need for further investigation using a more comprehensive injury definition [15, 16]. Apophyseal injuries may also lead to persistent symptoms during periods of rapid growth and high training loads, potentially affecting participation and performance in adolescent athletes [4, 9, 13]. However, the long‐term consequences of these injuries, including the proportion of athletes who develop chronic symptoms or discontinue sport participation, remain unknown.

The aim of this study was to investigate the prevalence, incidence, and characteristics of apophyseal injuries in the lower extremities in male adolescent academy players.

## 2. Methods

### 2.1. Study Design and Participants

This is a prospective cohort study in Norwegian male adolescent academy football players during the 2022 season. Prior to the season, all 66 male academy players, aged 12 to 19, were invited to participate, and 58 consented. During the 42‐week study period, five players transferred to other clubs and one player withdrew from the study. These players were included in the data analysis for their active weeks but excluded from the remaining weeks. No players were excluded on a minimum response rate cutoff. The study period was 42 weeks (February to December 2022), which is the regular football season in Norway. Baseline injury status was established for all participants at the start of the study going through an initial musculoskeletal screening and completing the first OSTRC‐H2 report.

### 2.2. Data Collection and Definitions

The players’ self‐reported health problems were monitored using the Oslo Sports Trauma Research Center Questionnaire on Health Problems (OSTRC‐H2) [15], completed weekly on their mobile phones through the Excellent Performance System (XPS) application. Reminders were sent to nonresponders once weekly. To handle repeated measurements of the same health problem, a single case was registered if symptoms persisted in the same anatomical area over consecutive weeks. Participants with missing weekly responses were excluded from the analysis for those specific weeks, and no imputation of missing data was performed. [16]. A health problem was defined as any physical complaint sustained during football activities, regardless of the need for medical attention or time‐loss [16, 17]. An apophyseal injury was defined when a player reported pain provoked by activity from the affected apophysis and tenderness upon palpation directly over the affected apophysis confirmed by a clinical examination [7, 8].

Health problems, including injuries and illnesses, were defined as any condition affecting a player’s physical, mental, or social well‐being [16]. Players were categorized as having a health problem if they recorded any symptoms in the questionnaire, or if they reported reduced training participation, training volume, or performance due to their health problem [16].

### 2.3. Clinical Examination

The clinical examination protocol included a thorough medical history, detailed clinical examination, and a number of clinical tests aimed at identifying apophyseal injuries in the lower extremities (Appendix 1). Examinations were performed by a sports physiotherapist (SR) with 4 years of clinical experience, following the standardized protocol developed in collaboration with the coauthors (TEA and JH). The clinical examination was conducted within 72 h of symptoms reported in XPS. Across all skeletal sites, a consistent diagnostic framework was systematically applied based on the underlying traction‐induced pathophysiology, in alignment with established clinical guidelines for apophyseal injuries [7]. Diagnoses were established when a player presented with localized point tenderness upon precise anatomical palpation directly upon the affected apophysis, combined with pain provocation during a resisted strength test of the muscle group inserting at that specific apophysis. This standardized approach was used for established diagnostic entities, including Osgood–Schlatter’s disease at the tibial tuberosity, Sinding–Larsen–Johansson syndrome at the inferior patellar pole, Sever´s disease at the calcaneal apophysis, and apophyseal injuries of the pelvis, including pubic apophysitis, AIIS apophysitis, ischium apophysitis, and iliac crest apophysitis. Diagnoses were established by the primary examiner (SR), with complex or prolonged cases quality‐assured by a senior clinician and coauthor with 10 to 30 years of clinical experience (JH and TEA). Additionally, MRI (Volumetric Interpolated Breath‐hold Examination [VIBE] protocol) confirmed the findings in selected cases.

### 2.4. Statistical Analysis

Descriptive analyses were performed using Microsoft Excel 365 (Version 2303). We calculated the prevalence of self‐reported health problems in OSTRC‐H2 and the prevalence of new apophyseal injuries, which were identified in the clinical examination. For both measures, the weekly prevalence was determined by dividing the number of new health problems or apophyseal injuries by the total number of completed OSTRC‐H2 questionnaires. To illustrate longitudinal variability, 95% confidence intervals (CI) for the weekly prevalence rates were calculated using standard proportion intervals and are visually presented as vertical error bars in Figure 1.

**FIGURE 1:**
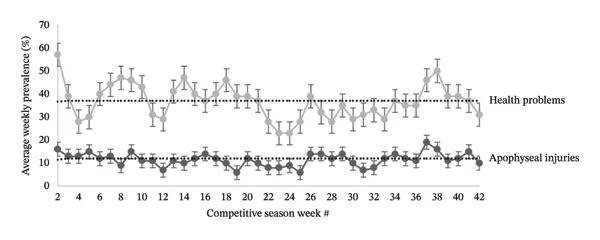
Overview of the average weekly prevalence (%) of all self‐reported health problems and clinically diagnosed apophyseal injuries to the lower extremities over the 42‐week‐season. The gray dotted line represents the mean of all self‐reported health problems captured via the OSTRC‐H2 questionnaire, while the black dotted line represents apophyseal injuries confirmed through a clinical examination. The vertical error bars represent 95% confidence intervals (CI).

The incidence of apophyseal injuries was calculated by determining the number of new cases per 1.000 h of exposure during training and matches. To avoid overestimation, repeated reports of symptoms in the same anatomical area were treated as a single injury case.

### 2.5. Patient and Public Involvement Statement

This project idea was developed by the authors. The author presented the idea to the male youth academy coaching staff, players, and their guardians in the club. The coaching staff played an active role in both planning and conducting the project. Their motivation was to document health problems within the academy in alignment with the Norwegian Academy Classification criteria for youth football development [18].

### 2.6. Ethics

This study involved human participants and complied to ethical standards by obtaining approval from the local ethics committee at the Norwegian School of Sport Sciences (NIH) on January 28th, 2022 (NIH Ethics Committee Approval Ref No. 217–270122), and from the Norwegian Center for Research Data (NSD) on February 4th, 2022 (NSD Project No. 734078). Written informed consent was obtained from participants aged 16 and older, while consent for players < 16 years was provided by their guardians before participation. It was clearly communicated that participation was voluntary and declining would not affect the players’ medical follow‐up or position in the academy. The study is described according to the STROBE statement checklist [19]. Personal data, including unique personal ID numbers, were anonymized and securely stored in a database at the NIH.

## 3. Results

### 3.1. Participants and Response to the Questionnaire

Of the 66 academy players invited, 58 accepted and were included in the study. During the 42‐week period, five transferred to other clubs, and one withdrew from the study due to the time commitment required for the weekly OSTRC‐H2 questionnaire, resulting in a total of 52 players included in the analyses.

We received 2011 responses from the questionnaires, resulting in an average weekly response rate of 91%. Demographic player characteristics are shown in Table 1.

**TABLE 1:** Demographic characteristics at baseline and weekly training exposure for each player group.

Group	Number of players (*n*)	Age (years)	Stature (cm)	Body mass (kg)	Body mass index	Weekly team football training exposure (hours)
U‐15	16	12.8 ± 0.6	160.9 ± 7.0	48.0 ± 8.2	18.4 ± 1.8	7.5 ± 1.3
U‐17	20	14.5 ± 0.6	167.6 ± 10.1	58.1 ± 13.4	20.8 ± 2.9	7.2 ± 0.8
U‐19	22	16.9 ± 0.9	178.1 ± 6.3	72.7 ± 6.7	22.9 ± 1.0	7.8 ± 0.6

*Note:* Age, stature, body mass, body mass index, and weekly team football training exposure; mean (± SD).

### 3.2. Prevalence and Incidence

The average weekly prevalence of self‐reported health problems was 37%, while the clinically examined and diagnosed apophyseal injuries to the lower extremities had an average weekly prevalence of 12%. The overall incidence rate for the apophyseal injuries was 4.6 per 1000 training and match hours. Figure 1 provides an overview of the average weekly prevalence of both health problems and apophyseal injuries, highlighting trends over one full season of 42 weeks.

### 3.3. Apophyseal Injuries

A total of 37 clinical examinations for suspected apophyseal injuries were carried out, resulting in 31 registered injuries in 21 players (36%). Twelve players had one, eight had two, and one had three apophyseal injuries. Seven types of apophyseal injuries were identified, with pubic apophysitis being the most common, with a total of 10 diagnosed cases (32%). Key data are shown in Figure 2.

**FIGURE 2:**
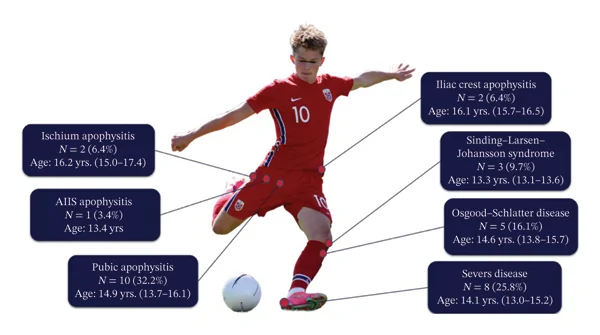
Overview of apophyseal injuries to the lower extremities, categorized by diagnosis, number of cases (*n*), percentage of the apophyseal injuries, and the age at the onset of injury (mean and range). Photo reproduced with permission from The Football Association of Norway.

### 3.4. Mode of Onset

Among the apophyseal injuries to the lower extremities, 77% had a gradual onset, 13% were classified as acute, and 10% involved recurrent sudden onset symptoms. Most injuries (81%) were reported during or after training, 13% during or after matches, and 6% were related to other sporting activities.

Most injuries occurred in the dominant leg (42%), bilateral involvement (39%), and the nondominant leg (19%). Moreover, 29% of the injuries occurred during the preseason period, while 71% during the competitive season.

### 3.5. Discussion

Our findings expand upon the current literature by providing a long‐term perspective on apophyseal injuries in youth football. The main findings were an average weekly prevalence of all health problems of 37% and an average weekly prevalence of clinically diagnosed apophyseal injuries of 12%. This study demonstrates that apophyseal injuries represent a significant burden in youth academy football, particularly during periods of rapid growth and high training loads.

Although apophyseal injuries are often considered self‐limiting conditions related to skeletal immaturity, they may still represent a substantial health problem in adolescent athletes [4–6, 13].

In the present cohort, a considerable number of players experienced clinically diagnosed apophyseal injuries during the season, and more than one‐third reported health problems at any given week. Persistent symptoms have been described in some athletes and may influence training participation and performance [9]. However, the mechanisms explaining why some apophyseal injuries become chronic are not well understood and may be related to factors such as high training loads during rapid growth periods, insufficient monitoring of early complaints, and participation in sports despite symptoms [8, 9].

### 3.6. Health Problems and Apophyseal Injuries: Prevalence and Incidence

The self‐reported prevalence of health problems in this study is comparable to a Norwegian study using identical method looking at high‐level youth athletes from a variety of both team sports and individual sports, reporting an average weekly prevalence of 43% [20].

To our best knowledge, this is the first study to report the weekly prevalence of clinically diagnosed apophyseal injuries, showing an average prevalence of 12%. Comparison with previous studies is challenging due to variations in methodology and injury definition. Materne et al. [21] (2022) reported that 84% of all injuries were apophyseal, considerably higher than our findings. This discrepancy may be explained by the fact that Materne et al. [21] (2022) reported the proportion of apophyseal injuries of all injuries in their cohort, while other studies have reported an overall seasonal prevalence between 4.5% and 14% [22–24]. An important difference might be that in these earlier studies they did not collect weekly injury data. While in this study, collection of weekly prevalence data provides a more precise picture of the real burden of these injuries over time, which is the key to understand the true extent and variation of apophyseal injuries in young football players.

In this study, we found an incidence of 4.6 apophyseal injuries per 1000 training and match hours which is in accordance with 4.5 per 1000 h reported by Materne et al. [21] (2022) for lower limb apophyseal injuries. Similarly, Read et al. [24] (2018) reported an incidence of 4.7 injuries per 1000 h. Earlier studies reported lower incidence rates from 0.4 to 1.0 [22, 23]. These findings suggest that the incidence of apophyseal injuries may have increased over time, although differences in study design, diagnostic criteria, and injury definitions should be considered when comparing studies across different periods.

### 3.7. Characteristics of Apophyseal Injuries

To date, there is no available literature reporting weekly clinical characteristics of apophyseal injuries in youth football. We found that 36.2% of all diagnosed injuries were apophyseal, which represents a considerably higher proportion than those previously reported by Gudelis et al. [9] (2022), showing seasonal prevalence ranging from 5.1% to 15.3% over seven seasons in the FC Barcelona football academy. Notably, while Gudelis et al. [9] documented apophyseal injuries affecting the ischiopubic ramus, the specific clinical diagnosis of pubic apophysitis was not uniquely isolated in their cohort. In contrast, we found a total of 10 players (32.2%) diagnosed with pubic apophysitis at a mean age of 14.9 years. This notable finding suggests that pubic apophysitis injuries may have been underreported or misclassified within broader pelvic categories in previous studies. We would argue that the standardized comprehensive clinical examination protocol to diagnose and categorize apophyseal injuries, particularly those affecting the pubic region, highlights the importance of specific attention to this area during clinical assessment of adolescent football players.

Previous studies predominantly identify Osgood–Schlatter’s disease and Sever’s disease as the most common conditions observed in youth football players [13, 22–26]. Whereas studies of pubic apophysitis is lacking, it is likely underreported. A study on Australian Football League players found that pubic apophysitis was present in 92% of AFL players experiencing groin pain [27]. This finding underline that the prevalence of pubic apophysitis in high‐demand sports, involving kicking, passing, and change of direction, might not be recognized and therefore often be misdiagnosed or underdiagnosed in youth football.

These discrepancies highlight the need for standardized definitions and methodologies in injury surveillance and reporting to enhance the comparability of the findings across studies.

Furthermore, it should be noted that the current cohort included players only up to 19 years of age. Since skeletal maturation of the pubic symphysis continues into the early twenties, the true prevalence of pubic apophysitis might be underestimated in this study [28].

Physiologically, rapid longitudinal bone growth during the pubertal growth spurt increases traction stress on immature apophyseal growth plates [6, 7]. To manage this risk, clubs should implement routine growth monitoring to identify periods of heightened vulnerability [4, 10]. Given that the U14 age cohort demonstrated the highest risk of apophyseal injuries, medical staff should prioritize monitoring during this developmental window [4]. Finally, enhancing targeted education for coaches, players, and parents is essential to foster early reporting of gradual‐onset symptoms, allowing for timely training volume modifications before clinical progression occurs.

### 3.8. Methodological Consideration

High compliance (91.4%) with the OSTRC‐H2 questionnaire showed strong player motivation and participation, in alignment with previous similar studies [29]. This is particularly positive in youth players given the risk of study fatigue due to weekly questionnaires. Given the high average weekly response rate, the potential influence of missing data on the results was considered minimal.

One obvious limitation of this study is the one male academy and one whole season only cohort. While this does limit the generalizability of the findings to other male academies in different countries and levels of play or to female academies, the high compliance still allows for a relatively robust analysis within the study’s context. Furthermore, focus on a single academy allowed for a more detailed insight into injury trends in this specific environment, providing an understanding that may not be as apparent in broader studies but still be relevant also in other contexts.

The researcher held a dual role as both head academy physiotherapist and primary investigator (SR), which may have introduced ethical challenges related to voluntary participation in the study [30, 31]. On the other hand, the dual role also enabled a detailed and contextually informed investigation, ensuring accurate classification and reporting of injuries in a familiar clinical setting. To address the risk of potential observer bias associated with this setup, a strict clinical examination protocol with objective criteria was made, and senior coauthors reviewed complex diagnostic cases to ensure consistency. Additionally, potential reporting bias was minimized by systematically validating the weekly self‐reported OSTRC‐H2 health data against these objective clinical examinations.

A strength of the current study was the specific standardized clinical examination protocol and thorough classification of lower extremity apophyseal injuries. While some conditions, such as pubic apophysitis, lack established diagnostic criteria, the standardized protocol ensured consistency and reliability of the examinations.

While advanced imaging such as MRI is often considered a gold standard for identifying apophyseal injuries [7], its routine application in a Norwegian football academy setting is restricted by practical and economic factors. To further support and strengthen our diagnostic approach, radiological imaging utilizing an MRI VIBE protocol, was used to confirm pubic apophysitis in three prolonged cases, showing sufficient alignment with our clinical diagnostic framework. For the remaining cases, further imaging was not considered necessary due to milder classic symptoms, a rapid return to play, and a successful response to load management. Given that previous monitoring studies in youth football rarely require routine imaging [1–4], these findings may indicate that the clinical correlation between clinical presentation and MRI confirmation in selected cases support the use of clinical evaluation without the necessity for routine imaging. Therefore, we suggest that a thorough clinical examination, centered on detailed history taking, functional resistance testing, and careful palpation, provides a practical, cost‐effective alternative approach for identifying these injuries, which appears well‐suited for daily academy football and other elite sports’ settings [6, 9].

### 3.9. Clinical Implications

This study highlights the high burden of apophyseal injuries in a male youth football academy with pubic apophysitis being the most common diagnosis. To mitigate the consequences of apophyseal injuries, it is essential to improve education among medical staff, coaches, players, and guardians to recognize early signs, such as gradual onset pain and common symptom locations [32]. Monitoring growth is a simple and effective preventive measure [33], but early detection and recording of symptoms is probably as effective and less sophisticated.

At the same time, proper load management should be implemented, including individualized training programs and football activity modification [33]. In addition, make sure players are educated about the diagnosis, the prognosis, and pain expectations to increase awareness and compliance to optimize recovery and return to football.

### 3.10. Future Directions

Given the limited research on apophyseal injuries, specifically on pubic apophysitis, they seem to be not recognized and underdiagnosed both in football and other sports [27, 34]. Future research should investigate the long‐term consequences of these injuries, including the risk of persistent symptoms and the potential impact on continued sport participation. Regarding pubic apophysitis, further studies are required to provide a clear definition and classification. Furthermore, appropriate intervention studies on management of these injuries are warranted to implement better mitigation and secondary prevention strategies.

Additionally, future studies should investigate the diagnostic correlation between standardized clinical examinations and MRI findings in a dedicated cohort to formally evaluate the clinical necessity of routine advanced imaging for pelvic apophyseal injuries.

To ensure improved generalizability, a larger study over multiple seasons including different cohorts of players is needed to better understand the prevalence and impact of these injuries as well as injury mechanisms and risk factors [35].

Moreover, focus on the interaction between growth, maturity, gender, and injury risk to improve mitigation and prevention strategies is scarce and warrant further investigation [35].

Female youth players are underrepresented in research on apophyseal injuries despite higher lower‐extremity injury rates [36, 37]. Females have earlier growth spurt which may increase injury risk [38], but limited evidence hinders targeted interventions. Studies on female players, not just youth but also younger than 13 years of age, are a low‐hanging fruit for future studies on apophyseal injuries.

## 4. Conclusion

This study highlights the high prevalence and incidence of apophyseal injuries in high‐level male academy football players, with pubic apophysitis being the most common. The U‐15 players showed the highest prevalence, indicating a need for increased awareness in the younger age groups. There is a need for more precise definitions and classifications, focusing specifically on pubic apophysitis, to improve future treatment and management protocols which can mitigate unnecessary pain and frustration increasing youth athletes’ participation in football or other sports.

## Author Contributions

Simen Rygh was responsible for the study coordination, data collection, clinical examinations, data analysis, and drafting the original manuscript. Thor Einar Andersen and Joar Harøy contributed to the project planning, study design, development of the clinical examination protocol, quality assurance of complex diagnostic cases, and provided critical intellectual revisions to the manuscript throughout the writing process.

## Funding

Oslo Sports Trauma Research Center has been established at the Norwegian School of Sport Sciences through generous grants from the Royal Norwegian Ministry of Culture, the South‐Eastern Norway Regional Health Authority, the International Olympic Committee, the Norwegian Olympic Committee and Confederation of Sport, and Norsk Tipping AS.

## Disclosure

All authors reviewed and approved the final version to be submitted. This study was previously presented as a conference abstract at the IOC World Conference on the Prevention of Injury & Illness in Sport, Monaco, 2024, and published as an abstract in the *British Journal of Sports Medicine* (BJSM) [39].

## Conflicts of Interest

The authors declare no conflicts of interest.

## Supporting Information

Additional supporting information can be found online in the Supporting Information section.

## Supporting information


**Supporting Information** Strobe.

## Data Availability

The data supporting the findings of this study are available from the corresponding author upon reasonable request. The personal data from the participants were anonymized and stored securely using an institutional database and a dedicated secure drive at the Norwegian School of Sport Sciences. The data are not publicly available due to privacy and ethical restrictions regarding health information from minor participants.

## Appendix 1 Clinical Examination Protocol

Clinical Examination Protocol.

Master thesis.

«What is the extent of growth‐related injuries in the lower extremities in boys’ academy football players?»

Clinical examination.

**Examiner:** _______________________________________________________________________

**Player ID:** ________________________________________________________________________

**Date of first examination (DD/MM/YY):** ______/______/_______

Inspection:

**□** Hydrops **□** Rubor/color **□** Calor/temperature **□** Other? ____________________________

Pain location:

Where are you hurting?

**□** Hip/groin **□** Knee **□** Heel **□** Other? ____________________________________

**□** Right **□** Left **□** Both **□** Other? ____________________________________________

What date did the injury/worsening of symptoms occur?

Page (DD/MM/YY): ______/______/_______

Has the pain come on acutely or gradually over time?

**□** Acute **□** Gradual **□** Acute worsening of symptoms that came gradually

Did the injury/worsening of symptoms occur in:

□ Football training □ Football match.

What match? (opponent and date): _____________________________________________________

Which minute of the match (approximately)? _____________________________________________

**□** Other activity _____________________________________________________________________

Describe in more detail how the injury/worsening of symptoms occurred?

_________________________________________________________________________________

_________________________________________________________________________________

_________________________________________________________________________________

For ALL types of damage. How does the player describe his pain?

**□** Sharp/stabbing **□** Throbbing **□** Murmuring **□** Tingling.

**□** Electrical **□** Numb **□** Burning **□** Other:

What movements and activities provoke the pain?

_________________________________________________________________________________

_________________________________________________________________________________

_________________________________________________________________________________

Has the player injured himself in the past?

**□** Yes **□** No.

What injuries? ______________________________________________________________________

Has the player had surgery?

**□** Yes **□** No.

What was the operation? _____________________________________________________________

Does the player have any other diseases?

**□** Yes **□** No.

What diseases? _____________________________________________________________________

Has the player had previous diseases?

**□** Yes **□** No.

What diseases? _____________________________________________________________________

Is the player on regular medication?

**□** Yes **□** No.

What medications? _________________________________________________________________________________

Number of weekly trainings:

Team training: _____

School training: _____

Personal training: _____

How much pain does the player have on a scale of 0–10?

Everyday life: _____

Before training: _____

During exercise: _____

After training: _____

Palpation of symptomatic structure: _____

Specific tests.

Go through *all the tests* for the right and left and put a cross where it fits based on whether the hip/groin, knee, and/or ankle should be examined. Examine the pain‐free side first. Categories for tests that are not looking for pain provocation are marked with positive or negative.

Pain categories:

1 Put a cross at **1** if the test reproduces the pain the player knows as **their pain**
**0+** Put a cross on **0+** if the test causes pain, but where this is **not** the player’s current pain
1 Put a cross at **0** if the test does not cause pain
